# Oral cavity-derived stem cells and preclinical models of jaw-bone defects for bone tissue engineering

**DOI:** 10.1186/s13287-023-03265-z

**Published:** 2023-03-16

**Authors:** Jie Zhao, Ying-Hui Zhou, Ya-Qing Zhao, Zheng-Rong Gao, Ze-Yue Ouyang, Qin Ye, Qiong Liu, Yun Chen, Li Tan, Shao-Hui Zhang, Yao Feng, Jing Hu, Marie Aimee Dusenge, Yun-Zhi Feng, Yue Guo

**Affiliations:** 1 Department of Stomatology, The Second Xiangya Hospital, Central South University, 139 Renmin Middle Road, Changsha, 410011 Hunan China; 2 National Clinical Research Center for Metabolic Diseases, Hunan Provincial Key Laboratory of Metabolic Bone Diseases, and Department of Metabolism and Endocrinology, The Second Xiangya Hospital of Central South University, Changsha, 410011 Hunan China

**Keywords:** Oral cavity-derived stem cells, Models, Jaw-bone defects, Bone tissue engineering

## Abstract

**Background:**

Jaw-bone defects caused by various diseases lead to aesthetic and functional complications, which can seriously affect the life quality of patients. Current treatments cannot fully meet the needs of reconstruction of jaw-bone defects. Thus, the research and application of bone tissue engineering are a “hot topic.” As seed cells for engineering of jaw-bone tissue, oral cavity-derived stem cells have been explored and used widely. Models of jaw-bone defect are excellent tools for the study of bone defect repair in vivo. Different types of bone defect repair require different stem cells and bone defect models. This review aimed to better understand the research status of oral and maxillofacial bone regeneration.

**Main text:**

Data were gathered from PubMed searches and references from relevant studies using the search phrases “bone” AND (“PDLSC” OR “DPSC” OR “SCAP” OR “GMSC” OR “SHED” OR “DFSC” OR “ABMSC” OR “TGPC”); (“jaw” OR “alveolar”) AND “bone defect.” We screened studies that focus on “bone formation of oral cavity-derived stem cells” and “jaw bone defect models,” and reviewed the advantages and disadvantages of oral cavity-derived stem cells and preclinical model of jaw-bone defect models.

**Conclusion:**

The type of cell and animal model should be selected according to the specific research purpose and disease type. This review can provide a foundation for the selection of oral cavity-derived stem cells and defect models in tissue engineering of the jaw bone.

**Supplementary Information:**

The online version contains supplementary material available at 10.1186/s13287-023-03265-z.

## Introduction

Jaw-bone defects can result from a various of congenital and acquired factors, such as cleft lip and palate [1], congenital developmental deformities and trauma [2, 3], jaw tumors [4], or tooth extraction [5]. Delayed healing or nonunion of jaw-bone defects can lead to masticatory difficulties, esthetic problems, and language dysfunction. The repair of jaw-bone defects is a challenging problem for stomatologists [6].

Various methods are used for jaw-bone regeneration: autogenous/allogenic/xenogeneic bone transplantation, distraction osteogenesis, and guided bone regeneration. Autogenous bone transplantation is the “gold standard” due to its osteogenesis, bone induction, and capacity for bone conductivity [7], but has the shortcomings of donor-site infection, pain, and limited available bone [8, 9]. Allogenic and xenogeneic bone transplantation can elicit the complications of disease transmission and an immunogenic response. Distraction osteogenesis can lead to a series of complications: fracture of basal bone and transport segment, tilting of segments, change of the distraction vector, breakage of the distractor, soft-tissue problems, and severe mechanical problems. Besides, removal of the internal retractor necessitates a second procedure, which reduces patient compliance considerably [10, 11]. Guided bone regeneration is used widely in the repair of small defects of the jaw. It has some disadvantages, such as the requirement for a stable barrier membrane and a new creative space during the procedure, potential complications, and relatively high costs [12, 13]. Also, it takes time for the bone powder used in guided bone regeneration to be replaced by natural bone, which affects orthodontic tooth movement [14]. Therefore, the above-mentioned methods cannot fully meet the needs for reconstruction of jaw-bone defects, and engineering of bone tissues provides new and feasible treatment options [15].

Bone tissue engineering (BTE) connects engineering, material science, biology, and medicine [16]. Suitable scaffold materials and feasible seed cells are important components [17] for BTE. Stem cells (SCs) have the capacity for multipotent differentiation and self-renewal. They are available from the dental tissues [18], bone marrow [19], umbilical-cord blood [20], and adipose tissue [21]. They are the most widely used seed cells due to their key role in bone formation, accessibility, and expansion potential [22]. SCs transplanted into a defect site can differentiate into osteoblasts and mimic the biological process of natural bone development, thereby inducing bone regeneration [23]. There are many types of SCs in the oral cavity, and several kinds of defect models are used to study the repair of jaw defects. Different SCs derived from the oral cavity have different characteristics and differentiation potential, and various defect models are suitable for multifarious types of bone defect-based diseases. Hence, selection of the correct SCs type and model of jaw-bone defects is important for BTE. However, selection of appropriate SCs and defect models is a difficult problem for scholars due to the special structure and physiologic characteristics of the oral and maxillofacial region.

This paper reviews the advantages and disadvantages of oral cavity-derived SCs and preclinical models of jaw-bone defects. Firstly, we introduced the characteristics of oral tissue, including teeth, jaws, and periodontal tissue. In order to better understand oral-derived SCs and how to select cells in oral bone tissue engineering, we introduced the tissue origin, surface markers, multi-directional differentiation capability of the oral-derived SCs and, their therapeutic significance in bone defect healing in oral tissue engineering. Then, the modeling methods, advantages, disadvantages, and application range of jaw defect models to provide a better reference for the selection of animal models in oral bone tissue engineering.Data were gathered from PubMed searches and references from relevant studies using the search phrases “bone” AND (“PDLSC” OR “DPSC” OR “SCAP” OR “GMSC” OR “SHED” OR “DFSC” OR “ABMSC” OR “TGPC”); (“jaw” OR “alveolar”) AND “bone defect.” We screened the retrieved search results and selected those articles that focus on “bone formation of oral cavity-derived SCs” and “jaw bone defect models.” Only published data were included in this review. In the final section, we discussed the transformation and prospect of BTE from basic research to clinical application.

## Characteristics of teeth, jaw bone, and periodontal tissue

**Fig. 1:**
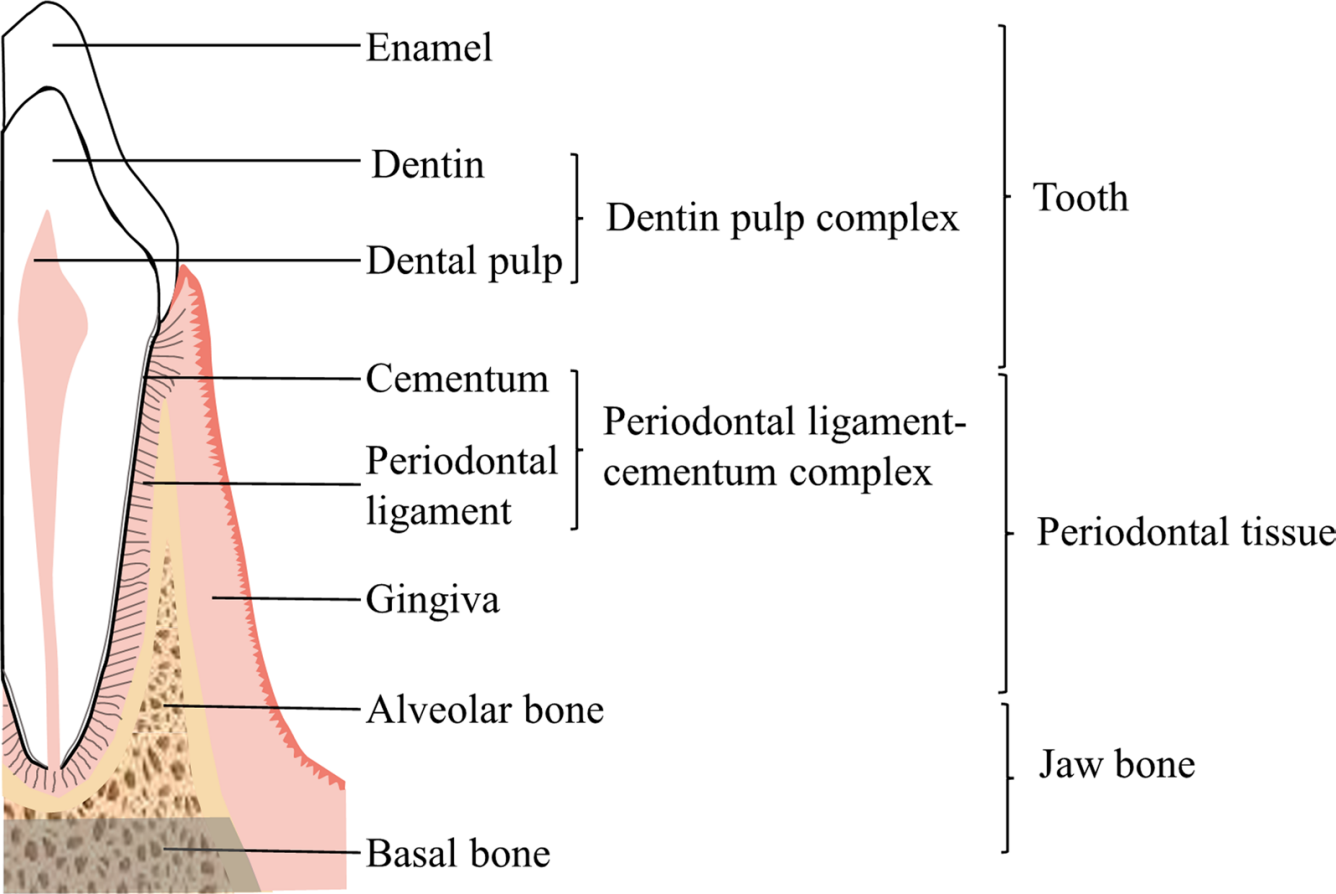
Composition of the jaw bone and periodontal tissue. A tooth is composed of enamel, dentin, dental pulp, and cementum, in which dentin and dental pulp form the dentin–pulp complex. Periodontal tissue is composed of cementum, the periodontal ligament, gingiva, and alveolar bone, in which cementum and the periodontal ligament form the periodontal–ligament cementum complex. The jaw bone is divided into alveolar bone and basal bone. Schematic created with Microsoft PowerPoint

As an important part of the maxillofacial region, the jaw bone has crucial role in maintaining the stability of the oral system, mastication, and facial appearance. The maxillofacial region consists of the maxilla and mandible. The mandible and maxilla still have some different characteristics. The canine and premolar regions of the maxilla have the maximum bone density, whereas the maxillary tuberosity has the minimum bone density. Cortical bone density in the mandible is higher than that in the maxilla and increased gradually from the incisor area to the retromolar area [24]. According to attachment/non-attachment of teeth, the maxilla and mandible are divided into alveolar bone and basal bone (Fig. 1). Basal bone is weighty and has a supporting role, and it is denser and less porous than alveolar bone. As the most important part of the skeletal system, the alveolar bone is closely related to the development, eruption, movement, masticatory function, and exfoliation of teeth. The change of alveolar bone reflects bone remodeling in the oral region [25].

Teeth attached to the jaw are not only organs that perform masticatory functions directly, but also play an important part in assisting pronunciation, speech, and maintaining facial coordination and beauty. Teeth consists of dental pulp, cementum, enamel, and dentin [26, 27] (Fig. 1). As the toughest tissue in the human body, enamel bears direct masticatory pressure. Dentin forms the main body of the tooth, and the dental pulp within it forms dentin. From the viewpoint of anatomy, physiology, and embryology, dentin and dental pulp develop from dental papilla and belong to one system, so they are called the “dentin–pulp complex.” The cementum located on the tooth root surface attaches the tooth tightly to alveolar bone through collagen fibers contained within it [28].

The jaw and teeth have been described from an anatomic viewpoint, but, in terms of clinical application, periodontal tissue is more closely related to diseases. Periodontal diseases are inflammatory diseases caused by pathogenic bacteria that bring harm to periodontal tissue, which includes bone and soft tissue that supports the teeth [29]. Periodontal tissues play an important part in the normal function of teeth, including gingiva, alveolar bone, cementum, and periodontal ligament (Fig. 1). The periodontal ligament, as a link between alveolar bone and cementum, can resist and regulate the pressure on teeth during mastication [30]. The periodontal ligament and cementum constitute the periodontal ligament–cementum complex, which facilitates tooth-alveolar bone relative motion. The strength of the gingiva determines the strength and firmness of teeth [31].

## SCs in the oral cavity with osteogenic potential

**Fig. 2:**
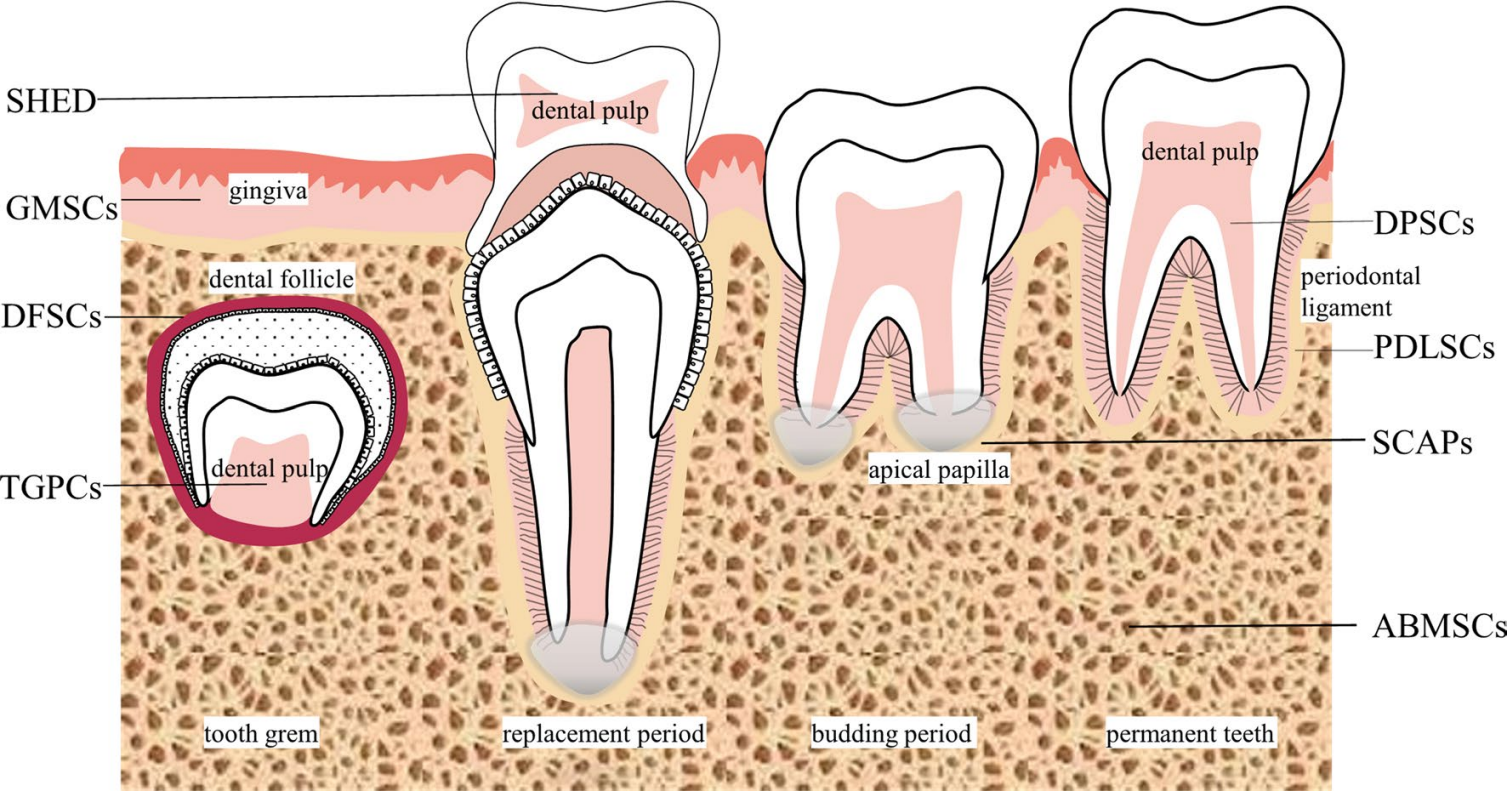
Stem cells in the oral cavity. Oral cavity-derived stem cells: PDLSCs, DPSCs, SCAPs, GMSCs, SHED, DFSCs, ABMSCs and TGPCs. PDLSCs are isolated from the periodontal ligament. DPSCs are isolated from dental pulp. SCAPs are isolated from the apical papilla of an impacted tooth. GMSCs are isolated from the gingiva. SHED are isolated from the pulp of deciduous teeth. DFSCs are isolated from dental follicles. ABMSCs are isolated from alveolar bone. TGPCs are isolated form the tooth germ. PDLSCs, periodontal ligament stem cells; DPSCs, dental pulp stem cells; SCAPs, stem cells from the apical papilla; GMSCs, gingiva‐derived mesenchymal stem cells; SHED, stem cells from exfoliated deciduous teeth; DFSCs, dental follicle progenitor cells; ABMSCs, alveolar bone‐derived mesenchymal stem cells; TGPCs, tooth germ progenitor cells. Schematic created with Microsoft PowerPoint

Mesenchymal SCs (MSCs) are at the forefront of new therapeutic approaches because they can differentiate into a variety of cell types and renew themselves [32]. Various studies have shown that MSCs have a wide application prospect in BTE. The most commonly used MSCs are bone marrow MSCs (BMSCs) [33], oral-derived SCs, and adipose-derived MSCs (ASCs) [34]. The Committee on Mesenchymal Stem cells and tissue Stem cells of the International Society of Cell Therapy has proposed a minimum standard for the definition of MSCs: 1) When MSCs are cultured under standard culture conditions, it is adherent to the wall. 2) MSCs express cluster of differentiation (CD)90, CD73, and CD105, but do not express CD11b or CD14, CD19, CD34, CD45, CD79a, and surface molecules of HLA-DR. 3) In vitro, MSCs exhibit plasticity for osteogenesis, chondrogenesis, and adipogenesis. [35, 36]. Oral-derived SCs share the described common features with MSCs isolated from other sources [34]. SCs in the oral cavity include alveolar bone‐derived MSCs (ABMSCs), dental follicle progenitor cells (DFSCs), dental pulp SCs (DPSCs), gingiva-derived MSCs (GMSCs), periodontal ligament SCs (PDLSCs), SCs from the apical papilla (SCAPs), SCs from exfoliated deciduous teeth (SHED), and tooth germ progenitor cells (TGPCs). There are a few small differences in the markers expressed by different oral-derived SCs, but they are generally similar. Detailed marker expression profiles for each of the oral-derived SCs are displayed in Table 1. Oral SCs are named according to their different sources [37]. The sources of oral-derived SCs are shown in Fig. 2.

**Table 1:** Isolation methods and characteristics of oral cavity-derived SCs

SCs	Positive markers	Negative markers	Sources	Time of ALP staining	Time of calcium accumulation	Markers of osteogenic differentiation	References
PDLSCs	CD166, CD146, CD106, CD105, CD90, CD73, CD59, CD44, CD29, CD13, CD10, CD9, SSEA4, 3G5, STRO-1	CD45, CD34, CD31, CD14	Third molar	7 days 14 days	4 weeks	ALP, OCN, MEPE, BSP, TGFβR1, RunX_2_ calcium accumulation	[41, 45–48]
DPSCs	CD271, CD166, CD146, CD106, CD105, CD90, CD73, CD59, CD49, CD44, CD29, CD13, CD10, CD9	CD133, CD117, CD45, CD34, CD31, CD24, CD19, CD14	Third molar	40 days 60 days	30 days 3 weeks 6 weeks	ALP, OCN, ON, BSP, DSPP, BAP, BMP2, calcium accumulation	[63–65, 68, 77]
SCAPs	CD166, CD146, CD106, CD105, CD90, CD73, CD61, CD56, CD51, CD44, CD29, CD24, CD13	CD150, CD117, CD45, CD34, CD18, CD14	Impacted third molar	3 days 4 weeks	7 days 14 days 4 weeks	ALP, calcium accumulation	[85, 87, 90–94]
GMSCs	CD166, CD146, CD106, CD105, CD90, CD73, CD44, CD29,	CD117, CD45, CD34	Gingival tissues	2 weeks	4 weeks	ALP, OCN, calcium accumulation	[59, 98, 101, 103]
SHED	CD166, CD146, CD105, CD90, CD73, CD56, CD44, CD29, CD13	CD45, CD43, CD34, CD19, CD14, CD11b	Deciduous incisors	1 days 3 days 7 days 14 days 4 weeks	14 days 4 weeks	RUNX2, ALP, OCN, MEPE, BSP, DSPP, Osterix, calcium accumulation	[109, 112, 114, 115]
DFSCs	D271, CD166, CD106, CD105, CD90, CD73, CD59, CD53, CD44, CD29, CD13, CD10, CD9, electron dense granular material	CD133, CD45, CD34, CD31, CD14	Third molar at root-developing stage	2 weeks 15 days	2 weeks 15 days 5 weeks	COL1, OCN, BSP, nestin, Notch1, electron-dense granular material	[124, 125]
ABMSCs	CD166, CD146, CD105, CD90, CD73, CD71, CD44, CD29, CD13,	CD45, CD34, CD31, CD19, CD14, CD11b	Alveolar bone	28 days	21 days 28 days	ALP, OCN, BSP, OP, calcium accumulation	[130–132]
TGSCs	CD166, CD106, CD105, CD90, CD73, CD44, CD29	CD133, CD45, CD34, CD14	Impacted third molar	14 days	10 days 2 weeks	ALP, COL1, DMP1, DSP, klf4, c-myc, oct4, nestin, NS, sox2, vimentin, hTERT, BMP2, BMP7, calcium accumulation	[143–145, 147, 148]

SC, stem cells; ABMSCs, alveolar bone‐derived mesenchymal stem cells;ALP, alkaline phosphatase; BAP, bone alkaline phosphatase; BMP, bone morphogenetic protein; BSP, bone sialoprotein; CD, cluster of differentiation;COL1, collagen type-I; DFSCs, dental follicle progenitor cells; DMP1, dentin matrix protein 1; DPSCs, dental pulp stem cells; DSPP, desmoplakin; GMSCs, gingiva‐derived mesenchymal stem cells; hTERT, human telomerase reverse-transcriptase; klf4, kruppel-like factor 4; MEPE, matrix extracellular protein; Notch-1, notch receptor 1; NS, nucleostemin; OCN, osteocalcin; ON, osteonectin; OP, osteopontin; PDLSCs, periodontal ligament stem cells; RUNX2, runt-related transcription factor 2; SCAPs, stem cells from the apical papilla; SHED, stem cells from exfoliated deciduous teeth; SSEA4, stage-specific embryonic antigen-4; TGFβR1, transforming growth factor-β receptor type I; TGPCs, tooth germ progenitor cells

Oral cavity-derived SCs form the main components of teeth (e.g., dentin [38] and cementum), and dental structural complexes (e.g., dentin–pulp complex [39], periodontal ligament–cementum complex [40, 41]), and the bone tissue formation is another one of their important functions [42]. Compared with BMSCs, oral cavity-derived SCs have a higher proliferation rate, are easier to obtain [43], and are very promising sources of SCs for alveolar bone regeneration. The alveolar bone marrow, periosteum, dental tissues, and gingival tissue are available SCs sources. In terms of the means of acquisition, dental tissues can be less invasive compared to BMSCs because they are “medical waste” which makes them less ethically problematic. And these tissue-derived SCs can be easily amplified from human body with minimal discomfort [9, 44]. The common isolation methods of oral cavity-derived SCs are listed in Additional file 1: Table S1. This information could provide a basis and methods for BTE and the clinical application of SCs.

### PDLSCs

PDLSCs are multipotent postnatal SCs contained in the periodontal ligament. PDLSCs were first isolated and amplified in vitro by Seo and colleagues in 2004 [45]. PDLSCs express the cell-surface molecules (CD)66, CD146, CD106, CD105, CD90, CD73, CD59, CD44, CD29, CD13, CD10, CD9, 3G5, stage-specific embryonic antigen-4 (SSEA4), and STRO-1, but not CD45, CD34, CD31, or CD14 [41, 45–48]. PDLSCs have immunosuppressive properties and possess low immunogenicity [49, 50]. PDLSCs have the ability to differentiate into adipocytes, osteoblasts, collagen-forming cells, and cementoblast-like cells under specific culture conditions.

PDLSCs were mainly isolated from the human periodontal ligaments. Some studies have also isolated PDLSCs from the periodontal ligament of animals, such as mice [51], rats [52], and rabbits[53]. Enzyme digestion is the most common method to obtain PDLSCs. The periodontal ligament is separated gently from the surface of the middle root section of the third molar and then digested in a solution of collagenase type-I and dispase for 1 h at 37 °C. A single-cell suspension was obtained by passing cells through a strainer [41, 45–48]. Several studies have been conducted on PDLSCs' ability to induce osteogenic differentiation in vitro and osteogenesis in vivo. Seo and colleagues revealed that, following a 4-week osteogenic induction, immunohistochemistry and western blotting showed that PDLSCs release Alizarin Red S (ARS) staining and alkaline phosphatase (ALP) demonstrated that PDLSCs formed small circular nodules, which indicated calcium deposition [46]. Similarly, Feng and colleagues found deposition of calcium nodules after osteogenic induction of PDLSCs for 4 weeks according to ARS staining [45]. Some scholars inoculated PDLSCs on different fibrous membranes to induce osteogenic differentiation for 7 days and 14 days, and the activity of ALP increased to varying degrees [48]. However, Kato and coworkers showed that PDLSCs can undergo osteogenic differentiation without osteogenic induction [54]. In vivo experiments in rats [41], beagle dogs [55], miniature pigs [56], and humans [45] have demonstrated that transplantation of PDLSCs can regenerate cementum, the periodontal ligament, and alveolar bone. In situ tissue engineering, whereby the periodontal ligament is implanted into the periodontal defects of rats for 1, 2, 4, and 8 weeks, revealed that PDLSCs regenerated the cementum–ligament–bone complex at the defect site [57]. PDLSCs not only have a good ability for bone regeneration but also are an important cell source for periodontal tissue regeneration. PDLSCs can form cementum-periodontal ligament complex in vivo and have the potential to form new periodontal attachment and repair periodontal defects [46, 48, 57, 58]. PDLSCs may also provide a new and reliable strategy for periodontal ligament formation in biological root regeneration. A vitamin C-induced PDLSCs sheet was covered on a root-shaped hydroxyapatite-tricalcium-phosphate (HA/TCP) scaffold, and then, the scaffold was implanted into a freshly formed jaw-bone socket. Following implantation for six months, PDLSCs could form a functional periodontal ligament-like structure in the process of biological root regeneration.[47]**.** Tendon regeneration is another application of PDLSCs. Encapsulated PDLSCs, which develop based on transforming growth factor-β3-loaded RGD-coupled alginate microspheres, were subcutaneously implanted into immunocompromised mice for 4 weeks and showed a stronger tendon regeneration ability than BMSCs or GMSCs [59]. At present, PDLSCs have been primarily used for tissue regeneration in humans. In a clinical trial, autologous PDLSCs cell membrane was used to treat 3 patients with periodontitis, which found that the periodontal tissue was improved and cementum and periodontal ligament formation could be seen around the cell membrane[60]. In another clinical trial, after using autologous PDLSCs cell patch for 6 months, the probing depth, imaging bone height, and clinical attachment level of 10 patients with periodontitis were significantly improved, which further confirmed the safety and efficacy of autologous PDLSCs cell patch for long-term treatment[61]. However, Chen et al. found no significant difference alveolar bone height between the autologous PDLSCs cell patch treatment group and the control group [62].

### DPSCs

DPSCs are a colony of cloned and rapidly proliferating cells isolated from adult dental pulp. They were first extracted from tooth pulp tissues through enzymatic digestion [63]. DPSCs express the surface markers CD271, CD166, CD146, CD106, CD105, CD90, CD73, CD59, CD49, CD44, CD29, CD13, CD10, and CD9, but not CD133, CD117, CD45, CD34, CD31, CD24, CD19, or CD14 [64–66]. Under specific induction conditions, DPSCs can undergo odontogenesis [67], adipogenesis, and myogenesis. Without pre-induction, DPSCs can also differentiate toward odontogenic and adipogenic pathways [68].

Due to the advantages of easy accessibility, high proliferation capacity, and easy extraction, DPSCs have been suggested as a therapy for bone defects in tissue engineering [66, 69]. In most studies, DPSCs were obtained from the pulp tissue of permanent teeth, deciduous teeth and tooth germ in humans. There are also a few studies obtained DPSC from animals, including mice [70, 71], rats [72, 73], and rabbits [74]. DPSCs can be obtained by enzyme digestion. Tooth surfaces were cleaned and cut around the cementum–enamel junction using sterilized dental fissure burs to reveal the pulp chamber. Pulp tissue was gently separated from the crown and root and then digested in a solution of collagenase type-I and dispase for 1 h at 37 °C. A single-cell suspension was obtained by passing cells through a strainer.

Different scholars have different views on the ability of DPSCs to induce osteogenic differentiation in vitro and osteogenesis in vivo. Long-term culture (6 weeks) of DPSCs can lead to formation of ARS-positive condensed nodules with a high level of calcium [63]. ALP staining shows that ALP activity increases with increasing time after 3, 7, and 14 days of osteogenic induction by DPSCs, and ARS staining reveals formation of massive calcified nodules after 21 days of induction [75]. Some studies have demonstrated that the osteogenic differentiation ability of DPSCs is lower than that of PDLSCs and BMSCs [63]. After inducing the osteogenesis of DPSCs, PDLSCs, and GMSCs for 3 weeks, Gao and colleagues found that PDLSCs and GMSCs had higher ALP activity and denser calcified nodules than those of DPSCs [76]. Gronthos and colleagues induced DPSCs and BMSCs for 6 weeks and found that BMSCs formed more dense calcified nodules according to ARS staining [63].

In one study, DPSCs were inoculated on a “collagen sponge” scaffold, and the obtained biological complex could completely repair the defect in human mandibular alveolar bone [77]. Gendviliene and coworkers implanted DPSCs with different scaffolds into the calvarial defect of rats for 8 weeks, and histology and micro-computed tomography (CT) showed that more bone formed than the control group [69]. The proliferation, migration, and osteogenic ability of DPSCs are also related to immune regulation. Sonoda et al. found that IFN-γ enhanced T cell suppression and dentin formation of pulpitis-derived DPSCs by increasing telomerase activity [78]. After the treatment of interferon-γ, DPSCs showed enhanced proliferation and migration but reduced osteogenic/odontogenic differentiation, which may be related to the MAPK and nuclear factor (NF)-κB signaling pathways [79]. In addition to good bone regeneration ability, some studies have shown that DPSCs can regenerate cementum, bone, and periodontal ligament in vivo [80–82].

DPSCs are also used for the regeneration of dentin and dental pulp. In tissue immunocompromised mice, DPSCs grafts could produce a dentin-like structure surrounded by human odontoblast-like cells and pulp-like interstitial [83]. A root-shaped scaffold of HA/TCP containing DPSCs was covered by a vitamin C-induced PDLSCs sheet and implanted into a freshly formed jaw-bone socket transplanted into a recently created jaw-bone implant socket, and led to regeneration of pulp-like structures while producing functional biological roots [47]. DPSCs were transplanted subcutaneously into immunodeficient mice and could form a dental pulp–dentin complex at 3 weeks [68]. In terms of clinical application, Chu et al. implanted collagen matrix scaffold with DPSCs into the extraction fossa of mandibular wisdom teeth. The implantation of DPSCs can make the blood vessels evenly distribution and increase the bone mineral density and alveolar septum of the new bone in the extraction fossa, thus effectively reducing the alveolar bone resorption [84].

### SCAPs

SCAPs are a stem-cell population separated from the apical papilla of human teeth and were isolated for the first time by Sonoyama and colleagues [85]. Compared with DPSCs, SCAPs have a higher mineralization potential and proliferation rate and express MSC markers, including CD166, CD146, CD106, CD105, CD90, CD73, CD61, CD56, CD51, CD44, CD29, CD24, and CD13, but not CD150, CD117, CD45, CD34, CD18, or CD14 [85–87]. After induction, in vitro cultured SCAPs can differentiate into adipogenic, neurogenic, odontogenic, and osteoblastic cells.

After tooth extraction, the apical papilla and SCAPs can be easily isolated by separating the tissue at the tips of the developing roots by tweezers [85]. The current main source of SCAPs is the apical papilla of human teeth, and only a few studies have isolated them from the apical papilla of rat teeth [88, 89]. Enzyme digestion is a widely used method to obtain SCAPs. Root apical papilla was gently separated from the surface of the root, then minced, and digested in a solution of collagenase type-I and dispase for 30 min at 37 °C. A single-cell suspension of SCAPs was obtained by passing cells through a strainer [85, 87, 90–94].

The time of osteogenic differentiation in the study of osteogenic differentiation and bone formation of SCAPs is different. Zhou and colleagues cultured SCAPs under osteogenic conditions for 7 days, and ARS staining revealed formation of calcified nodules [95]. Some studies have shown that ALP was present after 3 days of culturing SCAPs, and calcium nodules were formed on 14 days [94]. Sonoyama and colleagues found that SCAPs require osteogenic induction for 4 weeks to form ARS-positive mineralized nodules [85]. With regard to bone formation in vivo, Li and collaborators injected overexpressed secreted frizzled related protein 2 (SFRP2) and normal SCAPs into the periodontal defects of miniature pigs for 4 weeks. They found that the probing depth, attachment loss, and gingival recession improved, and the amount of newly formed bone increased according to clinical assessment and CT, which indicated that SCAPs could mediate bone regeneration in periodontitis [96]. Some scholars have transplanted a SCAPs–HA complex into nude mice subcutaneously and found that SCAPs formed bone/dentin-like mineralized tissue at 8 weeks [92]. In addition, periodontal tissue regeneration is also an application field of SCAPs. SCAPs can form periodontal ligaments and can be served as seed cells for the regeneration of periodontal tissue in vivo [85].

SCAPs also have a key role in the formation of dentin and pulp tissue. In one study, SCAPs treated with epigallocatechin-3-gallate had a higher proliferation rate, mineral deposition, and ALP activity, and higher expression of odontogenic/osteogenic markers, including bone sialoprotein and collagen type-1, than SCAPs without treatment with epigallocatechin-3-gallate. Those data demonstrated that epigallocatechin-3-gallate promoted the odontogenic/osteogenic differentiation of SCAPs and could be used in regenerative dentistry [87]. Implantation of SCAPs into the root canal can lead to formation of dentin-like mineralized tissues. Mineral trioxide aggregate (MTA)-treated SCAPs were transferred into the root canal and implanted into the renal capsule of rats. Dental pulp-like structures containing dentin and odontoblast-like cells formed between soft tissue and MTA compared with the control group [93]. SCAPs can be used for regeneration of pulp nerves because they can elicit neurogenic differentiation. In neural‑induction medium, SCAPs can differentiate into neurogenic cells in vitro [91]. SCAPs were inoculated into the pulp cavity of human teeth after pulp removal and then implanted subcutaneously on the dorsal region of rabbits: dentin-like and pulp-like tissue was formed at 4 months [90].

### GMSCs

Zhang and coworkers isolated GMSCs from human gingival tissue for the first time in 2009 [97]. GMSCs expressed the MSC-associated markers CD166, CD146, CD106, CD105, CD90, CD73, CD44, and CD29, but not CD117, CD45, or CD34 [97–100]. GMSCs are readily accessible, have immunomodulatory and anti-inflammatory functions, and can undergo multipotent differentiation (e.g., adipocytes, odontoblasts and osteoblasts [101]).

The main source of GMSCs is the lamina propria gingival tissue in human, and it has also been isolated from mouse gingival tissue [102]. GMSCs are obtained by enzyme digestion. Gingival tissue was washed twice in phosphate-buffered saline. After removal of the epithelial layer, tissue was minced into 1–3 mm^2^ fragments and incubated in mixture of 0.1% dispase and 0.2% collagenase type-IV for 15 min at 37 °C. The first digested cell suspensions were discarded, and then, the tissues were incubated in 0.2% trypsin solution for 5, 10, and 15 min at 37 °C. All cell fractions were collected and seeded with complete alpha-modified minimal essential medium [59, 98, 101, 103].

Some studies have focused on the abilities of osteogenic differentiation and bone-tissue formation of GMSCs. Dong and colleagues cultured GMSCs under osteogenic conditions for 35 days; they found strong osteogenic potential with heavy deposition of minerals according to ARS staining [104]. Zhang and coworkers also found GMSCs could form ARS-stained positive nodules after 4 weeks of osteogenesis induction [97]. Two weeks after osteogenic induction, ALP staining of GMSCs was positive [98]. GMSCs showed a medium-level potential of osteogenic and adipogenic differentiation between those of PDLSCs and DPSCs [76]. GMSCs that were cultured with the osteogenic medium on the HA/TCP implants were blended with collagen gel and subcutaneously transplanted into the back of immunocompromised mice. High levels of type I collagen, osteocalcin, and osteopontin (OPN) expression in the transplant demonstrated the ability of GMSCs for bone regeneration in vivo [97]. GMSCs cultured on collagen gel were transplanted into the calvarial and mandibular defects of Sprague–Dawley rats. Two months after transplantation, the rapier speed of bone defect in the GMSCs implants group was faster than that of the gel control group lacking GMSCs, indicating that GMSCs can promote the healing of calvarial defects and mandibular wounds. Also, histomorphology and fluorescence imaging revealed that the freshly formed bone in the healing tissues was originated from GMSCs [98]. Hence, GMSCs could not only carry out ectopic osteogenesis, they also promoted the healing of jaw defects. In addition, GMSCs may stimulate osteogenesis by modulating immune cells. Zhao et al. found that GMSCs can stimulate MC3T3-E1 cells to differentiate into osteoblasts by inhibiting the function of activated T-cells through up-regulating IL-10 and down-regulating TNF-α and IL-1β. [105]. Furthermore, GMSCs can be employed to regenerate periodontal tissues, which can rebuild periodontal ligament, bone, and cement in areas with periodontal defects [103, 106].

In addition to bone formation, GMSCs are used to generate gingival tissue. Some scholars have found that GMSCs separated from inflamed gingival tissue have the same ability of adipogenic, osteogenic, and chondrogenic differentiation as that of healthy gingival tissue in vitro, and the same capacity to form connective tissue-like structures similar to normal gingival tissue [99]. Those findings suggest that GMSCs have a wide range of sources.

### SHED

SHED is a group of SCs isolated obtained from the residual dental pulp of exfoliated deciduous teeth. SHED express the surface markers CD166, CD146, CD105, CD90, CD73, CD56, CD44, CD29, and CD13, but not CD45, CD43, CD34, CD19, CD14, or CD11b [107–109]. SHED can be amplified in vitro and have the ability to differentiate into odontoblasts, vascular endothelial cells [110], adipocytes, smooth muscle cells [111], neural cells, and osteoblasts [112].

The residual pulp of exfoliated deciduous teeth is the reliable source of SHED, and no research has shown that SHED can be obtained from animals[113]. Enzyme digestion is the most common method to obtain SHED. Normal exfoliated human deciduous incisors were collected from 7–8-year-old children. Pulp was separated from a remnant crown and then digested in a solution of collagenase type-Iand dispase for 1 h at 37 °C. A single-cell suspension was obtained by passing cells through a strainer [114, 115].

Miura and coworkers cultured SHED under osteogenic conditions They found that ALP activity increased with the prolongation of induction time on 1, 3, 7, and 14 days of culture [109]. Some scholars have cultured SHED under osteogenic conditions for 14 days and found formation of calcified nodules according to ARS staining [109]. Miura and colleagues cultured SHED for 4 weeks; ARS staining showed formation of mineralized nodules in the induction, and immunohistochemical staining demonstrated that ALP was present [112]. Some studies have focused on the bone formation elicited by SHED in vivo. Seo and colleagues transplanted SHED/HA-TCP implants into the calvarial defects of immunocompromised mice, and found that SHED could repair the defects more quickly with substantial bone formation compared with the control group (HA/TCP carrier transplant without SHED) [115]. The deciduous teeth derived SHED was implanted into mandibular defects of miniature pig. The authors found that the SHED/β-TCP-treated group had faster repair with formation of many new bones than the control group in which only β-TCP scaffolds were implanted at 6 months [116]. Implantation of SHED into the periodontal defects of miniature pigs demonstrated that SHED could repair the soft-tissue and hard-tissue defects caused by periodontitis at 12 weeks [117]. After 32 days of subcutaneous implantation of SHED/PLLA-scaffolds in immunodeficient mice, SHED can differentiate into odontoblasts and form tubular dentin; they can differentiate into functional odontoblasts and form tubular dentin [118]. SHED can also affect bone volume by immunomodulation. Some studies have found that SHED can inhibit the expression of inflammatory factors INF- γ and TNF- α to reduce the production of osteoclasts, thus increasing the production of new attachment of periodontal ligament and alveolar bone volume [119, 120]. A few studies showed that SHED has a certain application value in periodontal regeneration and successfully achieved the regeneration of periodontal tissue in animal models [121].

In addition to the above-mentioned SHED-mediated regeneration of alveolar bone, the periodontal ligament, cementum, and dentin, regeneration of dental pulp is another application of SHED. SHED were transplanted into the minipigs’ empty root canals; 3 months later, dental pulp tissue with complete length was formed, and immunofluorescence staining confirmed that the dental pulp tissue arose from SHED [122].

### DFSCs

A dental follicle is an ectomesenchymal tissue surrounding the tooth germ in development. The SCs and directed progenitor cells or progenitor cells in dental follicles are called DFSCs. They express CD271, CD166, CD106, CD105, CD90, CD73, CD59, CD53, CD44, CD29, CD13, CD10, and CD9, but cannot express CD133, CD45, CD34, CD31, or CD14 [123–125]. DFSCs can differentiate into cementoblasts, osteoblasts, and periodontal ligament cells during tooth development and have the potential for osteogenic, chondrogenic, and adipogenic differentiation in vitro [125]. DFSCs are a heterogeneous population that exhibit various phenotypes. In 18 clones obtained from a single DFSC, only three clones were amplified for > 5 generations after 90–95 days of culture. Further study showed that the single DFSC and three clones had different characteristics: proliferation and apoptosis rate, differentiation characteristics, and lifespan [126].

DFSCs are mainly separated from dental follicle of impacted third molars in humans. DFSCs are easily obtained because third molar extraction is minimally invasive and harmful to healthy dentition. There are also some studies that choose to obtain DFSCs from the dental follicle tissues of rats [127] and mice [51]. The cell attachment method is employed to isolate DFSCs isolated by adherence to plastic from freshly extracted dental follicle tissues. A small number of single dental follicle tissue cells attached to the plastic surface and grew as fibroblastic cells. Non-adherent cells were removed by changing the medium [124, 125].

Some scholars have focused on the application of DFSCs in osteogenesis. Rezai-Rad and colleagues induced DFSCs for 2 weeks [123], whereas Guo and coworkers induced DFSCs for 15 days [124]: ALP-positivity and calcified nodules were confirmed by ALP staining and ARS staining, respectively. After induction of DFSCs osteogenesis for 5 weeks, Morsczeck and collaborators discovered formation of ARS-positive nodules [125]. A DFSCs scaffold was implanted into the craniofacial defects of rats. New bone was formed in the bone defects implanted with DFSCs-scaffold at 4 and 8 weeks, but not in the scaffold controls without DFSCs [123]. There are little data on the role of DFSCs in periodontal regeneration. Sowmya et al. implanted hydrogel scaffolds combined with human DFSCs into the rabbit maxillary periodontal defect model and found that the formation of cementum, alveolar bone, and periodontal membrane tissue increased [128].

DFSCs can also be used for regeneration of dentin and roots. DFSCs induced by a dentin matrix (TDM) differentiated into odontoblasts, expressed bone sialoprotein, osteocalcin, osteopontin, collagen type-I, and ALP and could regenerate intact prefabricated dentin in vivo. Guo and colleagues implanted a TDM with DFSCs in the omental pouches of adult rats for 2 weeks. They found that DFSCs could regenerate dentin [129]. DFSCs combined with TDM transplantation into the alveolar fossa could form root-like tissues with positive dental pulp markers (dentin matrix acidic phosphoprotein 1, dexamethasone sodium phosphate) and periodontal tissue (collagen type-I, scleraxis) after 4 weeks, thereby indicating the success of root regeneration [126].

### ABMSCs

ABMSCs are isolated from human alveolar bone marrow. They have the capacity for osteogenesis, adipogenesis, and chondrogenesis. ABMSCs can be acquired during implant surgery [130]. The karyotypes of ABMSCs are normal up to 30 population doublings, with significant cell senescence beginning after 35 population doublings [131]. ABMSCs have the surface markers of CD166, CD146, CD105, CD90, CD73, CD71, CD44, CD29, and CD13, but not CD45, CD34, CD31, CD19, CD14, or CD11b [75, 131–133].

The source of ABMSC can be human, rats [134, 135], and mice [136]. Since human ABMSC can be isolated from medical waste generated during implantation or surgery [137], the most common source is still human. The common methods employed to obtain ABMSCs are cell adhesion and enzyme digestion. The method of cell attachment involves placing bone marrow tissue directly into a culture medium to obtain adherent ABMSCs [132]. In the enzyme digestion method, the obtained trabecular bone grafts were chopped into small pieces in phosphate-buffered saline with 2% fetal bovine serum and then digested with collagenase type-I for 30 min at 37 °C. All cells were filtered through a strainer to produce a single-cell suspension [138].

Some researches have concentrated on the capacity of ABMSCs to elicit osteogenic differentiation and bone tissue formation. After 7 and 14 days of osteogenic induction, ALP staining showed that ALP was present at day-3 of osteogenic induction [75]. Matsubara and colleagues detected expression of ALP mRNA on day-28 [132]. Studies have shown that mineralized nodules are present according to ARS staining in cultured ABMSCs after 14 days and 21 days of osteogenic induction [75, 132]. Compared with BMSCs extracted from ilium, Matsubara and coworkers found that ABMSCs had a similar osteogenic ability and slightly lower ability of chondrogenesis and adipogenesis [132]. Qu and colleagues compared the osteogenic differentiation capacity of ABMSCs, PDLSCs, DPSCs, and DFSCs. They found that ABMSCs had the strongest ability for osteogenic differentiation. Consistent with that finding, Liu and collaborators discovered that ABMSCs had higher expression of osteogenic gene markers and mineral deposition on day-7 and day-14 of osteogenic induction, respectively [139]. You-Young and collaborators found that ABMSCs had a much greater capacity for mineralization compared with that of PDLSCs, DPSCs, and SCAPs [140].

ABMSCs have a stronger ability to differentiate into osteogenic cells in vitro and to form bone in vivo [139], and some studies have shown that ABMSCs can elicit ectopic osteogenesis. ABMSCs are not used in the repair of jaw defects. ABMSCs could promote the formation of ectopic-bone with vascularized tissue and trabecular bone following subcutaneous transplantation into mice at 4 weeks [131]. ABMSCs were transplanted using a microporous biphasic calcium phosphate carrier into the subcutaneous pocket of immunodeficient mice. Histology showed formation of more new bone tissue and higher collagen content of bone than the scaffold control group at 8 weeks [141]. Therefore, ABMSCs could be employed in the repair of jaw defects. ABMSCs have a certain application prospect in periodontal regeneration, which can regenerate the defect area by forming new bone, cementum tissue, and periodontal ligament-like fibers [142].

### TGPCs

TGPCs are a group pf SCs in the dental mesenchyme of the tooth germ in the third molar at late bell stage. They were separated from the molar mesenchyme by enzymatic digestion [143, 144]. TGPCs are positive for the MSC markers of CD166, CD106, CD105, CD90, CD73, CD44, and CD29, and negative for CD133, CD45, CD34, and CD14 [143, 145, 146]. Compared with human embryonic SCs, TGPCs express transcription factors that are essential for re-programming adult cells to induce pluripotent SCs, such as sox2, c-myc, and Kruppel-like factor 4 [145]. Under specific conditions, TGPCs can differentiate into adipogenic, neurogenic, and osteogenic cells, odontoblasts [145], and hepatocytes [144]. They can be cryopreserved, and the cryopreserved resuscitated cells can form new bones under the skin of immunocompromised rats [143].

The third molar tooth germs of humans are the source of TGPCs. For preventative reasons, the third molar tooth germs are usually removed and discarded during orthodontic treatment. Thus, TGPCs have a wide range of clinical sources. Cell adhesion and enzyme digestion are common methods to obtain TGPCs. In the cell attachment method, an entire tooth germ tissue is minced into small pieces and then transferred into plates containing a culture medium, and unattached cells are discarded by changing the culture medium [145, 147]. The enzyme digestion method involves digesting the entire tooth germ tissue with collagenase and shaking for 30 min at 37 °C. A pellet is obtained by centrifugation and resuspension in medium and then placed in a dish for primary culture [143, 144].

Several researches have concentrated on the capacity of TGSCs to elicit osteogenic differentiation in vitro and bone tissue formation. After two weeks of culture in osteogenic medium, the ALP mRNA expression and ALP activity of TGPCs increased according to polymerase chain reaction and ALP staining [147], and the formation of calcified nodules increased according to ARS staining [143, 144]. Osteogenesis was induced by TGPCs at 7, 14, and 21 days; the degree of mineralization increased with an increase in induction time and reached a peak on day-21 [146]. After induction for 1, 4, 7, 14, and 21 days, ALP staining of TGPCs showed that ALP activity reached a peak at day-7 [146]. The transplantation of TGPCs with a combination of polyethylene glycol-based hydrogel and biphasic calcium phosphate scaffolds can promote jaw-bone regeneration of pig [148]. At present, no research has confirmed that TGPCs can be used for periodontal tissue regeneration.

## Preclinical models of jaw-bone defects and its application

**Fig. 3:**
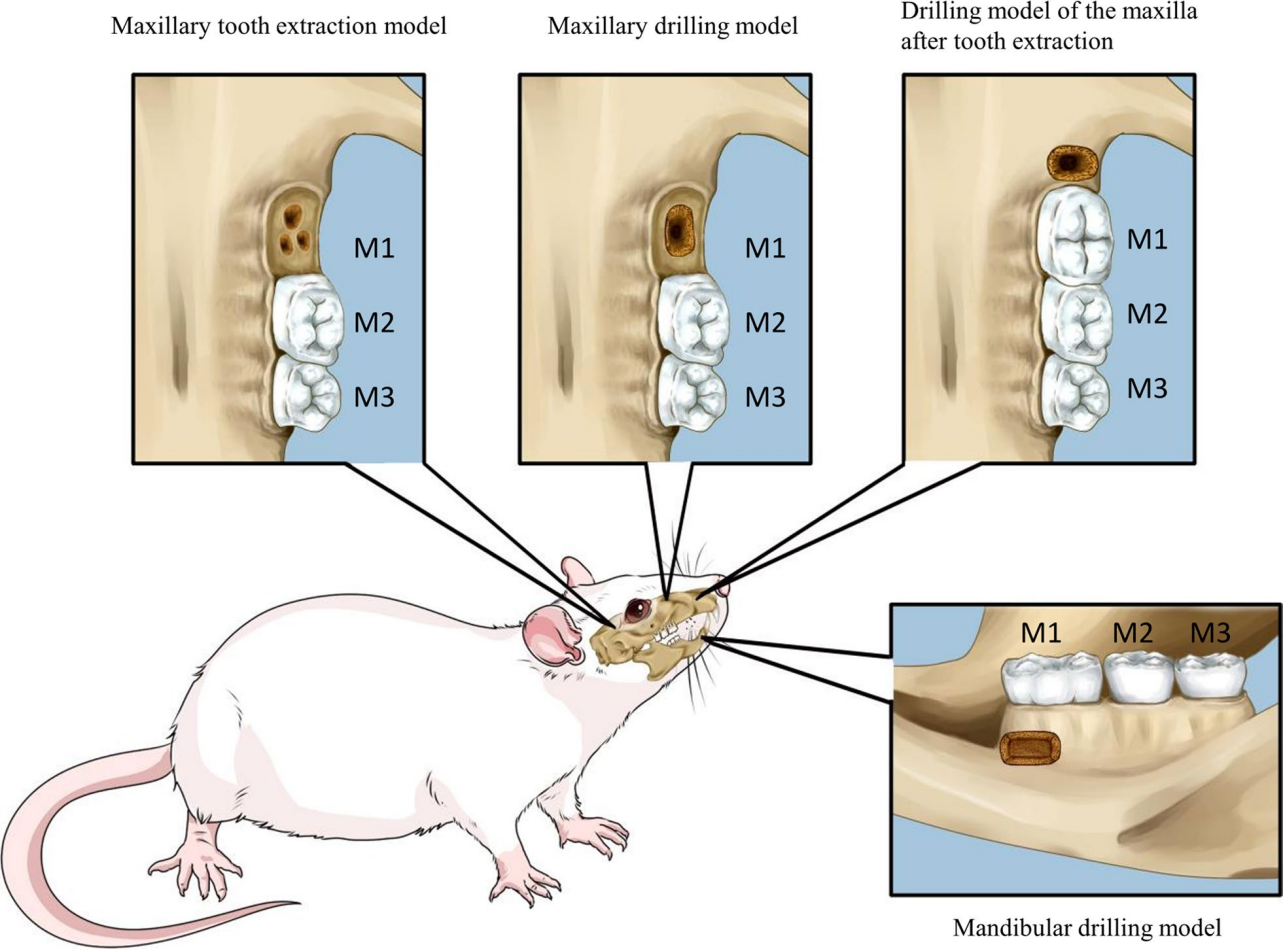
Jaw defect models in rats and mice: maxillary tooth extraction model; maxillary drilling model; drilling model of the maxilla after tooth extraction; mandibular drilling model. The maxillary tooth extraction model is established by extraction of the maxillary first molar. The maxillary drilling model is established by drilling near the first molar of the maxilla. The drilling model of the maxilla after tooth extraction is established by drilling after extraction of the first molar. The mandibular drilling model is established by drilling in the body of the mandible. Schematic created with Microsoft PowerPoint

Animal experiments can build a “bridge” between basic research and clinical application [149]. A jaw defect model in animals is used widely to explore the influencing factors of jaw defect healing and to find appropriate treatment methods to accelerate bone formation and healing speed. Various scenarios (tooth extraction, trauma, congenital deformities, periodontal disease) can cause jaw defects in different areas, tissues, and to different degrees. Preclinical models of jaw-bone defects include the tooth extraction model, drilling model, and post-extraction drilling model (Fig. 3 and Table 2), but none of them can completely simulate the defects caused by all diseases. Therefore, in animal experiments, different preclinical models of jaw-bone defects should be chosen according to the diseases necessitating treatments.

**Table 2:** Modeling methods and application of preclinical models of jaw-bone defects

Type of model	Position	Age of animals	Modeling methods	Duration of observation	Application	References
*Rat*						
Maxillary tooth extraction model	First molar	4 weeks	A dental probe was used to delicately separate the gingivae surrounding the right maxillary first molar. After the extraction of maxillary right first molar, inter-radicular septa were removed using a round bur while irrigating with saline	4 weeks	Healing of an alveolar bone defect after tooth extraction	[153]
Maxillary drilling model	Mesial position of the buccal side of the first molar	–	In close proximity to the gingival sulcus of first molar buccal, a relieving incision about 1 cm was made. After elevating the mucosal flap, a 3 × 1 × 1 mm defect was made by drilling the alveolar bone with a round bur while irrigating with saline. After the operation, the flap was restored and the wound was sutured with 5–0 surgical sutures	0, 4, 8 and 12 weeks	Bone defect healing	[155]
	Mesial–lingual side of the first molar	6 weeks	On the mesial–lingual side of the maxillary first molar, a defect was created by using a round bur of 3 mm diameter. Drilling the alveolar bone intermittently to prevent bone necrosis	8 weeks	Bone defect healing	[156]
Drilling model of the maxilla after tooth extraction	First molar	10–12 months	First, bilateral first molars were removed. Then, the extraction sockets were enlarged by a dental bur with a 2 mm diameter while irrigating with saline. The sockets were extended to a critical size of 2 mm in depth and 2 mm in diameter	0, 4 weeks	Alveolar-fossa healing	[158]
	Right first molar	10–12 months	First, the maxillary right first molar extracted. Then, from the maxillary right second molar’s cement–enamel junction, the extraction sockets were enlarged to defects with a 2 mm depth by using a dental bur with a 1.4 mm diameter while irrigating with saline. Finally, cyanoacrylate adhesive was used to seal the wound	0, 7, 14, and 28 days	Healing of an alveolar bone defect after tooth extraction	[159]
Mandibular drilling model	Mandibular body	6–8 weeks	An anteroposterior skin incision was done in the left orofacial location to reveal the underlying mandible. A mandible body defect of 5 × 2 × 1 mm was created by using a dental drill with a 1 mm diameter while irrigating with saline. After the operation, the wounds were sutured with 5–0 surgical sutures	8 weeks	Repair of mandibular defects	[98]
*Mouse*						
Maxillary tooth extraction model	First molar	10 weeks	Without fracturing the tooth or alveolar bone, the first maxillary molars of the right were extracted	0, 1, 2, 3, 4, 5, and 6 weeks	Healing of an alveolar bone defect after tooth extraction	[154]
Maxillary drilling model	First molar	6–7 weeks	Under sterile circumstances, a little incision was created in the premaxillary bone's mucosa and the soft tissues were raised. In the midline of each premaxilla, just posterior to the upper incisor, a defect with a 1.2 mm diameter was created by using a surgical trephine powered by a low-speed dental engine	30 days	Treatment of maxillary alveolar cleft	[157]
Drilling model of the maxilla after tooth extraction	First molar	6–7 weeks	First, the upper first molar was removed using dental forceps. Then, the extraction socket was widened and a four-wall bone defect was created by using a dental handpiece and tiny spherical bur	2, 4, and 6 weeks	Alveolar-fossa healing and the effect of graft material on orthodontic tooth movement	[14, 160]

Preclinical models of jaw-bone defects can employ large animals (e.g., miniature pigs [117], beagle dogs, goats and rabbits [150]) and small animals (rats and mice) [151]. Large animals have the disadvantages of expense (including feeding), inconvenient surgical procedures, and limited application. Compared with large animals, small animals are used more widely because of their easy ability, low cost, and convenient surgical procedures [152]. Therefore, we have reviewed preclinical models of jaw-bone defects in rats and mice.

### Maxillary tooth extraction model

The maxillary tooth extraction model can be established by extracting the maxillary first molar. Healing of alveolar bone defects can be investigated after tooth extraction. Nie and colleagues established a model by extracting the right maxillary first molar of rats to study the effect of a nano-HA mineralized silk fibroin scaffold with pre-osteoblasts on resorption of the alveolar ridge and bone formation. They found that the scaffold with pre-osteoblasts formed more new bone and reduced the height of alveolar bone resorption than the scaffold control group [153]. Mashimo and colleagues, by extracting the right maxillary first molar of mice, established a model to study the promotion of alveolar bone healing and bone-marrow formation after BMSC implantation into the extracted fossa. BMSCs were obtained from the femur and tibial bone marrow and transplanted immediately into the extraction alveolus. At 3 and 6 weeks after transplantation, bone formation in the alveolar fossa in the BMSC implantation group was significantly earlier than that in the control group without BMSCs [154].

### Maxillary drilling model

The maxillary drilling model is established by drilling holes in the proximal middle of the maxillary first molar with a low-speed dental handpiece. This model can be used not only for the study of bone defect healing, but also for treatment of the maxillary alveolar cleft.

Subramaniam and colleagues and Wen and coworkers studied bone defect healing by the maxillary drilling model in rats. An alveolar bone defect of 3 × 1 × 1 mm was constructed in close proximity to the maxillary first molar's buccal gingival sulcus of rats to form alveolar bone defects. After filling with HAP/CS/HA implants containing type-I collagenase (HAP/CS/HA-Col), micro-CT and histology revealed that HAP/CS/HA-Col had better formation of new bone and mature bone morphology than that of the control group implanted with HAP/CS/HA implants without type-I collagenase at 0, 4, 8, and 12 weeks after implantation [155]. In another study, a maxillary defect of diameter 3 mm was formed on the mesial lingual side of the maxillary first molar, and type-4 collagen A2 blended with bone powder and PDLSCs was transplanted to develop a strategy for repairing bone defects. They discovered that COL4A2 increased the osteogenic differentiation of PDLSCs by inhibiting the Wnt/-catenin pathway, which stimulate the production of more collagen fibrils and bone [156].

The maxillary drilling model in mice is used only for the treatment of maxillary alveolar clefts. Kawata and colleagues explored the therapeutic effect of an external callus on a maxillary alveolar cleft in a defect model at the midline of the premaxilla posterior to the upper incisor. They obtained an external callus comprising hyaline cartilage by tibial distraction osteogenesis with an external fixator and implanted it into the defects. They found that the external callus had bone formation and remodeling in its interior and promoted bone adhesion [157].

### Drilling model of the maxilla after tooth extraction

The drilling model of the maxilla after tooth extraction is established by extracting the first molar and drilling in the alveolar fossa. This model can be used to study healing of the alveolar fossa and the effect of graft material on orthodontic tooth movement.

After the bilateral maxillary first molars of rats had been extracted, Boda and colleagues enlarged the alveolar fossa to a critical defect of diameter 2 mm and depth 2 mm and implanted it with mineralized short fibers with and without heptaglutamate E7 domain-conjugated bone morphogenetic protein-2 peptides. They found that the volume of new bone and bone mineral density in these groups were threefold higher than those in the unfilled control group at 4 weeks [158]. Willett and collaborators established the drilling model of the maxilla after tooth extraction in rats by extracting the right maxillary first molar of. Then, they investigated the effect of simvastatin on bone preservation. After implanting a bovine bone mineralized matrix infused with simvastatin into the defect, they measured the width and height of the alveolar ridge, inflammation index, and bone turnover index at days 0, 7, 14, and 28. They found reduced inflammation and an increased alveolar ridge height compared with those in the bovine bone mineralized matrix-alone group and untreated control group [159].

Klein and colleagues created a defect of ~ 15 µl in the extracted fossa of the maxillary first molar and implanted bovine bone into it. They compared a bovine bone-implanted group with an untreated control group at 2, 4, and 6 weeks. They found that the xenogeneic bovine bone in the bovine bone-implanted group was not absorbed and could prevent tooth movement in the latter stage of orthodontics [14].

### Mandibular drilling model

The mandibular drilling model was established using a dental drill to create a mandibular body defect with a volume of 5 × 2 × 1 mm of Sprague–Dawley rats. Only one study has employed this model to investigate the role of GMSCs on mandibular defects repair.eGFP^+^ GMSCs/collagen gel matrix were implanted into the defect of mandibular. Histomorphology revealed that GMSCs were capable of repairing the mandibular defect, and fluorescence microscopy showed that the new formed bone originated from the GMSCs [98].

## Conclusion and future prospects for SCs in bone regeneration

In recent decades, great progress has been made in regenerative medicine, especially tissue engineering, which has been applied widely in several clinical scenarios. Tissue engineering based on SCs is a promising method to repair bone defects [161]. Basic research and clinical applications have demonstrated the advantages of oral cavity-derived SCs in jaw-bone regeneration [43, 85]. All oral cavity-derived SCs have osteogenic ability and can be used for the repair of jaw defects. In addition, PDLSCs are used mainly for periodontal tissue regeneration (periodontal ligament, alveolar bone, and cementum) and tendon tissue regeneration. DPSCs are employed mainly for the regeneration of alveolar bone, dentin, and dental pulp. SCAPs can form dentin-like and pulp-like tissues. GMSCs are often used to repair jaw-bone defects and regenerate periodontal ligament and dentin. SHED can promote the repair of cementum, alveolar bone, dentin, and the periodontal ligament. DFSCs can be used for the regeneration of dentin and roots.

Bone defect models also have important roles in BTE. Animal testing is a bridge between clinical applications and in vitro researches. The selection of animal models for in situ bone formation is closely related to specific clinical conditions [161]. Different animal models are suitable for the study of different diseases treatment. The maxillary models include the tooth extraction model, drilling model after tooth extraction model, and drilling model. They are more widely used than the mandibular model. There is only one model on the mandible, and that is the drilling model. The maxillary tooth extraction model can be employed to study the healing of alveolar bone defects after tooth extraction. The maxillary drilling model can be employed not only for the study of bone defect healing, but also for the treatment of maxillary alveolar clefts. The drilling model of the maxilla after tooth extraction can be used to study alveolar-fossa healing and the effect of graft material on orthodontic tooth movement. The mandibular drilling model is used to study the repair of mandibular defects. The types of cells and models should be selected according to the specific purpose of the study and the disease type. However, it is still unclear about the relationship between the size of model defects and oral-derived SCs. The elucidation of this relationship is of great guiding significance for future clinical treatment, although a great number of animal studies have provided excellent evidence that oral-derived SCs can be applied to regenerate bone tissue. Before this becomes a clinical reality, however, a number of crucial issues must be resolved, including figuring out which tissues can provide the most suitable cells, figuring out whether allogeneic cells can be used safely, comprehending the immunomodulatory and immunogenicity characteristics of oral-derived SCs, designing suitable delivery devices, weighing the cost/effectiveness, and designing methods to control the whole regeneration process. In conclusion, this review can provide a basis for the selection of oral cavity-derived SCs and defect models in tissue engineering of the jaw bone.

## Supplementary Information

**Additional file 1**. Isolation methods of oral cavity-derived SCs.

## Data Availability

Data sharing is not applicable to this article as no datasets were generated or analyzed during the current study.

## Abbreviations

ABMSCs: Alveolar bone-derived mesenchymal stem cells
ALP: Alkaline phosphatase
ARS: Alizarin Red S
BAP: Bone alkaline phosphatase
BMP: Bone morphogenetic protein
BSP: Bone sialoprotein
BTE: Bone tissue engineering
CD: Cluster of differentiation
COL1: Collagen type-I
DFSCs: Dental follicle progenitor cells
DMP1: Dentin matrix protein 1
DPSCs: Dental pulp stem cells
DSPP: Desmoplakin
GMSCs: Gingiva-derived mesenchymal stem cells
hTERT: Human telomerase reverse transcriptase
klf4: Kruppel-like factor 4
MEPE: Matrix extracellular protein
MSCs: Mesenchymal stem cells
Notch-1: Notch receptor 1
NS: Nucleostemin
OCN: Osteocalcin
ON: Osteonectin
OP: Osteopontin
PDLSCs: Periodontal ligament stem cells
RUNX2: Runt-related transcription factor 2
SCs: Stem cells
SCAPs: Stem cells from the apical papilla
SHED: Stem cells from exfoliated deciduous teeth
SSEA4: Stage-specific embryonic antigen-4
TGFβR1: Transforming growth factor-β receptor type I
TGPCs: Tooth germ progenitor cells
