# Supplementary material for: Oral cavity-derived stem cells and preclinical models of jaw-bone defects for bone tissue engineering
Source: Stem Cell Res Ther. 2023 Mar 16;14:39. doi: 10.1186/s13287-023-03265-z (PMC10022059; doi:10.1186/s13287-023-03265-z)
Supplement: Supplementary file 1 — Additional file 1. Isolation methods of oral cavity-derived SCs. [file 13287_2023_3265_MOESM1_ESM.docx]

**Supplement table 1**

Isolation methods of oral cavity-derived SCs

| SCs | Isolation methods |
| --- | --- |
| PDLSCs | The periodontal ligament was gently separated from the surface of the middle root section and then digested in a solution of collagenase type-I (3 mg/mL) and dispase (4 mg/mL) for 1 h at 37°C. A single-cell suspension was obtained by passing cells through a 70-μm strainer(1-5). |
| DPSCs | Tooth surfaces were cleaned and cut around the cementum–enamel junction using sterilized dental fissure burs to reveal the pulp chamber. Pulp tissue was gently separated from the crown and root, and then digested in a solution of collagenase type-I (3 mg/mL) and dispase (4 mg/mL) for 1 h at 37°C. A single-cell suspension was obtained by passing cells through a 70-μm strainer(6-10). |
| SCAPs | Root apical papilla from 18–20-years-old participants was gently separated from the surface of the root, then minced and digested in a solution of collagenase type-I (3 mg/mL) and dispase (4 mg/mL) for 30 min at 37°C. A single-cell suspension of SCAPs was obtained by passing cells through a 70-μm strainer(11-17). |
| GMSCs | Gingival tissue was washed twice in phosphate-buffered saline. After removal of the epithelial layer, tissue was minced into 1–3 mm2 fragments and incubated in mixture of 0.1% dispase and 0.2% collagenase type-IV for 15 min at 37°C. The first digested cell suspensions were discarded, and then the tissues were incubated in 0.2% trypsin solution for 5, 10, and 15 min at 37°C. All cell fractions were collected and seeded with complete alpha-modified minimal essential medium(18-21). |
| SHED | Normal exfoliated human deciduous incisors were collected from 7–8-year-old children. Pulp was separated from a remnant crown and then digested in a solution of collagenase type-I (3 mg/mL) and dispase (4 mg/mL) for 1 h at 37°C. A single-cell suspension was obtained by passing cells through a 70-μm strainer(22-25). |
| DFSCs | DFSCs isolated by adherence to plastic from freshly extracted dental follicle tissues. A small number of single dental follicle tissue cells attached to the plastic surface and grew as fibroblastic cells. Non-adherent cells were removed by changing the medium(26, 27). |
| ABMSCs | Method 1  The marrow of alveolar bone was opened during oral surgery to obtain marrow aspirates using routine syringes and needles without contamination by periodontal tissues. Bone marrow was placed in culture medium to obtain adherent cells(28, 29).  Method 2  Trabecular bone grafts were chopped into small pieces in phosphate-buffered saline with 2% fetal bovine serum and then digested with collagenase type-I for 30 min at 37°C. All cells were filtered through a 40-μm strainer to produce a single-cell suspension(30). |
| TGSCs | Method 1  Cell attachment: Entire tooth germ tissue was placed in 10-mm tissue-culture dishes with a sterile scalpel in Dulbecco’s modified essential medium. Tissue was minced into small pieces and transferred into six-well plates containing Dulbecco’s modified essential medium(31-33).  Method 2  Enzyme digestion: The entire tooth germ tissue was digested with 10 mL of collagenase (4 mg/mL) in PBS supplemented with 1 mM CaCl2, and shaken for 30 min at 37℃. Tissue was centrifuged at 400 ×g for 10 min at 4℃ to obtain a pellet, which was suspended in culture medium and placed in a 10-cm dish for primary culture(34, 35). |

SC, stem cells; ABMSCs, alveolar bone‐derived mesenchymal stem cells; DFSCs, dental follicle progenitor cells; DPSCs, dental pulp stem cells; GMSCs, gingiva‐derived mesenchymal stem cells; PDLSCs, periodontal ligament stem cells; SCAPs, stem cells from the apical papilla; SHED, stem cells from exfoliated deciduous teeth; TGPCs, tooth germ progenitor cells.

**References**

1. Seo BM, Miura M, Gronthos S, Bartold PM, Batouli S, Brahim J, et al. Investigation of multipotent postnatal stem cells from human periodontal ligament. Lancet. 2004;364(9429):149-55.

2. Feng F, Akiyama K, Liu Y, Yamaza T, Wang TM, Chen JH, et al. Utility of PDL progenitors for in vivo tissue regeneration: a report of 3 cases. Oral Diseases. 2010;16(1):20-8.

3. Hasegawa M, Yamato M, Kikuchi A, Okano T, Ishikawa I. Human periodontal ligament cell sheets can regenerate periodontal ligament tissue in an athymic rat model. Tissue Engineering. 2005;11(3-4):469-78.

4. Wei F, Song T, Ding G, Xu J, Liu Y, Liu D, et al. Functional tooth restoration by allogeneic mesenchymal stem cell-based bio-root regeneration in swine. Stem Cells Dev. 2013;22(12):1752-62.

5. Shang L, Liu Z, Ma B, Shao J, Wang B, Ma C, et al. Dimethyloxallyl glycine/nanosilicates-loaded osteogenic/angiogenic difunctional fibrous structure for functional periodontal tissue regeneration. Bioact Mater. 2021;6(4):1175-88.

6. d'Aquino R, De Rosa A, Lanza V, Tirino V, Laino L, Graziano A, et al. HUMAN MANDIBLE BONE DEFECT REPAIR BY THE GRAFTING OF DENTAL PULP STEM/PROGENITOR CELLS AND COLLAGEN SPONGE BIOCOMPLEXES. European Cells & Materials. 2009;18:75-83.

7. d'Aquino R, Graziano A, Sampaolesi M, Laino G, Pirozzi G, De Rosa A, et al. Human postnatal dental pulp cells co-differentiate into osteoblasts and endotheliocytes: a pivotal synergy leading to adult bone tissue formation. Cell Death Differ. 2007;14(6):1162-71.

8. Gronthos S, Mankani M, Brahim J, Robey PG, Shi S. Postnatal human dental pulp stem cells (DPSCs) in vitro and in vivo. Proc Natl Acad Sci U S A. 2000;97(25):13625-30.

9. Laino G, d'Aquino R, Graziano A, Lanza V, Carinci F, Naro F, et al. A new population of human adult dental pulp stem cells: A useful source of living autologous fibrous bone tissue (LAB). Journal of Bone and Mineral Research. 2005;20(8):1394-402.

10. Zhang W, Walboomers XF, Van Kuppevelt TH, Daamen WF, Van Damme PA, Bian Z, et al. In vivo evaluation of human dental pulp stem cells differentiated towards multiple lineages. Journal of Tissue Engineering and Regenerative Medicine. 2008;2(2-3):117-25.

11. Sonoyama W, Liu Y, Fang D, Yamaza T, Seo BM, Zhang C, et al. Mesenchymal stem cell-mediated functional tooth regeneration in swine. PLoS One. 2006;1(1):e79.

12. Liu Z, Lin Y, Fang X, Yang J, Chen Z. Epigallocatechin-3-Gallate Promotes Osteo-/Odontogenic Differentiation of Stem Cells from the Apical Papilla through Activating the BMP-Smad Signaling Pathway. Molecules. 2021;26(6).

13. Sequeira DB, Oliveira AR, Seabra CM, Palma PJ, Ramos C, Figueiredo MH, et al. Regeneration of pulp-dentin complex using human stem cells of the apical papilla: in vivo interaction with two bioactive materials. Clin Oral Investig. 2021.

14. Chen Q, Yuan C, Jiang S, Heng BC, Zou T, Shen Z, et al. Small molecules efficiently reprogram apical papilla stem cells into neuron-like cells. Exp Ther Med. 2021;21(6):546.

15. Yang H, Fan J, Cao Y, Gao R, Fan Z. Distal-less homeobox 5 promotes the osteo-/dentinogenic differentiation potential of stem cells from apical papilla by activating histone demethylase KDM4B through a positive feedback mechanism. Exp Cell Res. 2019;374(1):221-30.

16. Yan M, Wu J, Yu Y, Wang Y, Xie L, Zhang G, et al. Mineral trioxide aggregate promotes the odonto/osteogenic differentiation and dentinogenesis of stem cells from apical papilla via nuclear factor kappa B signaling pathway. J Endod. 2014;40(5):640-7.

17. Wang L, Yan M, Wang Y, Lei G, Yu Y, Zhao C, et al. Proliferation and osteo/odontoblastic differentiation of stem cells from dental apical papilla in mineralization-inducing medium containing additional KH(2)PO(4). Cell Prolif. 2013;46(2):214-22.

18. Wang F, Yu M, Yan X, Wen Y, Zeng Q, Yue W, et al. Gingiva-derived mesenchymal stem cell-mediated therapeutic approach for bone tissue regeneration. Stem Cells Dev. 2011;20(12):2093-102.

19. Kim D, Lee AE, Xu Q, Zhang Q, Le AD. Gingiva-Derived Mesenchymal Stem Cells: Potential Application in Tissue Engineering and Regenerative Medicine - A Comprehensive Review. Front Immunol. 2021;12:667221.

20. Moshaverinia A, Xu X, Chen C, Ansari S, Zadeh HH, Snead ML, et al. Application of stem cells derived from the periodontal ligament or gingival tissue sources for tendon tissue regeneration. Biomaterials. 2014;35(9):2642-50.

21. Yu X, Ge S, Chen S, Xu Q, Zhang J, Guo H, et al. Human gingiva-derived mesenchymal stromal cells contribute to periodontal regeneration in beagle dogs. Cells Tissues Organs. 2013;198(6):428-37.

22. Miura M, Gronthos S, Zhao M, Lu B, Fisher LW, Robey PG, et al. SHED: stem cells from human exfoliated deciduous teeth. Proc Natl Acad Sci USA. 2003;100.

23. Seo BM, Sonoyama W, Yamaza T, Coppe C, Kikuiri T, Akiyama K, et al. SHED repair critical-size calvarial defects in mice. Oral Dis. 2008;14.

24. Wang M, Li J, Ye Y, He S, Song J. SHED-derived conditioned exosomes enhance the osteogenic differentiation of PDLSCs via Wnt and BMP signaling in vitro. Differentiation. 2020;111:1-11.

25. Gronthos S, Zhao MR, Lu B, Fisher LW, Robey PG, Shi ST. SHED: Stem cells from human exfoliated deciduous teeth. Proceedings of the National Academy of Sciences of the United States of America. 2003;100(10):5807-12.

26. Morsczeck C, Gotz W, Schierholz J, Zellhofer F, Kuhn U, Mohl C, et al. Isolation of precursor cells (PCs) from human dental follicle of wisdom teeth. Matrix Biology. 2005;24(2):155-65.

27. Guo W, Chen L, Gong K, Ding B, Duan Y, Jin Y. Heterogeneous dental follicle cells and the regeneration of complex periodontal tissues. Tissue Eng Part A. 2012;18(5-6):459-70.

28. Matsubara T, Suardita K, Ishii M, Sugiyama M, Igarashi A, Oda R, et al. Alveolar bone marrow as a cell source for regenerative medicine: differences between alveolar and iliac bone marrow stromal cells. J Bone Miner Res. 2005;20(3):399-409.

29. Mason S, Tarle SA, Osibin W, Kinfu Y, Kaigler D. Standardization and safety of alveolar bone-derived stem cell isolation. J Dent Res. 2014;93(1):55-61.

30. Park JC, Kim JC, Kim YT, Choi SH, Cho KS, Im GI, et al. Acquisition of human alveolar bone-derived stromal cells using minimally irrigated implant osteotomy: in vitro and in vivo evaluations. J Clin Periodontol. 2012;39(5):495-505.

31. Yalvac ME, Ramazanoglu M, Rizvanov AA, Sahin F, Bayrak OF, Salli U, et al. Isolation and characterization of stem cells derived from human third molar tooth germs of young adults: implications in neo-vascularization, osteo-, adipo- and neurogenesis. Pharmacogenomics J. 2010;10(2):105-13.

32. Taşlı PN, Yalvaç ME, Sofiev N, Sahin F. Effect of F68, F127, and P85 pluronic block copolymers on odontogenic differentiation of human tooth germ stem cells. J Endod. 2013;39(10):1265-71.

33. Ramazanoglu M, Moest T, Ercal P, Polyviou Z, Herrmann K, Gurel Pekozer G, et al. The effect of polyethylenglycol gel on the delivery and osteogenic differentiation of homologous tooth germ-derived stem cells in a porcine model. Clin Oral Investig. 2020.

34. Ikeda E, Hirose M, Kotobuki N, Shimaoka H, Tadokoro M, Maeda M, et al. Osteogenic differentiation of human dental papilla mesenchymal cells. Biochem Biophys Res Commun. 2006;342(4):1257-62.

35. Ikeda E, Yagi K, Kojima M, Yagyuu T, Ohshima A, Sobajima S, et al. Multipotent cells from the human third molar: feasibility of cell-based therapy for liver disease. Differentiation. 2008;76(5):495-505.
